# Opioid-Induced Constipation in Patients with Cancer Pain in Japan (OIC-J Study): A Post Hoc Analysis

**DOI:** 10.3390/jcm10184193

**Published:** 2021-09-16

**Authors:** Akihiro Tokoro, Hisao Imai, Soichi Fumita, Toshiyuki Harada, Toshio Noriyuki, Makio Gamoh, Masaharu Okamoto, Yusaku Akashi, Yoshiyuki Kizawa

**Affiliations:** 1 Department of Psychosomatic Internal Medicine and Supportive and Palliative Care Team, National Hospital Organization Kinki-Chuo Chest Medical Center, Sakai 591-8555, Japan; tokoro.akihiro.qb@mail.hosp.go.jp; 2 Division of Respiratory Medicine, Gunma Prefectural Cancer Center, Ota 373-8550, Japan; 3 Department of Respiratory Medicine, Comprehensive Cancer Center, International Medical Center, Saitama Medical University, Hidaka 350-1298, Japan; 4 Department of Medical Oncology, Kindai University Nara Hospital, Nara 630-0293, Japan; souitifumita@hotmail.co.jp (S.F.); y-akashi@med.kindai.ac.jp (Y.A.); 5 Center for Respiratory Diseases, JCHO Hokkaido Hospital, Sapporo 062-8618, Japan; harada-toshiyuki@hokkaido.jcho.go.jp; 6 Department of Surgery, Onomichi General Hospital, Onomichi 722-8508, Japan; nori0509_0217@yahoo.co.jp; 7 Department of Medical Oncology, Osaki Citizen Hospital, Osaki 989-6183, Japan; m.gamoh-oc@h-osaki.jp; 8 Medical Affairs, Shionogi & Co., Ltd., Osaka 541-0045, Japan; mmm.okamoto@gmail.com; 9 Department of Palliative Medicine, Kobe University Graduate School of Medicine, Kobe 650-0017, Japan; ykizawa@med.kobe-u.ac.jp

**Keywords:** bowel function index, cancer pain, Japanese patients, observational study, opioid-induced constipation, Rome IV criteria

## Abstract

Opioid-induced constipation (OIC) can limit the clinical benefit of opioid treatment. This post-hoc analysis evaluated the association between the Rome IV diagnostic criteria and other measures for OIC, including the Bowel Function Index (BFI), correlation between demographics and OIC onset, impact of OIC on pain treatment, and impact of patient–healthcare professional (HCP) communication on patient satisfaction. Patients recorded bowel habits in paper diaries for 14 days following opioid initiation. Study-specific questionnaires were used to evaluate patient awareness of OIC and satisfaction. Patients were ≥20 years old, initiating strong opioid therapy for cancer pain, had an ECOG PS ≤ 2, and had no constipation (≥3 bowel movements within 7 days of enrollment). A total of 220 patients were enrolled. The sensitivity and specificity of BFI for identifying OIC were 81.2% and 54.7%, respectively. Age <65 versus ≥65 years (odds ratio (OR) = 0.510, 95% confidence interval (CI): 0.267–0.977) and the presence or absence of comorbidities (OR = 0.443, 95% CI: 0.221–0.885) were correlated with OIC onset. The proportion of inpatients with sustainable pain control at week 2 was similar in patients with or without OIC (60.0% vs. 67.2%, respectively). By patient assessment, there was a significant correlation between an adequate level of patient–HCP communication and satisfaction with OIC treatment (OR = 9.538 (95% CI: 1.577–57.681)). Using BFI to screen for OIC represents a valid approach in patients with cancer pain. Patient–HCP communication is essential for effective management of OIC in patients with cancer pain.

## 1. Introduction

Opioids are the standard of care for the treatment of moderate to severe cancer pain. However, while effective in pain management, opioids are associated with a number of gastrointestinal adverse events, including opioid-induced constipation (OIC), which can limit the clinical benefit of opioid treatment [1,2]. OIC is characterized by difficult-to-pass and hard stools, straining at defecation, and sensations of incomplete evacuation or anorectal obstruction after the initiation of opioid treatment [3,4]. Up to 94% of patients receiving opioids for pain experience OIC [5], which has been shown to be a significant patient burden [6]. In addition to the burden of physical symptoms, there is a substantial impact of OIC on activities of daily living, productivity, and relationships, and many patients report feelings of frustration, anxiety, and worry related to their OIC [6,7,8]. Evidence also suggests that patients find it difficult to manage pain relief and OIC symptoms [6], with some patients indicating that they do not adhere to their prescribed opioid regimen in order to manage their OIC [6,9]. Some studies have also demonstrated that patients believe that OIC interferes with their pain management [7,10].

Despite being a frequent occurrence in patients receiving treatment for pain, OIC has been shown to be poorly managed and remains underdiagnosed and undertreated [6]. Studies have shown that healthcare professionals (HCPs) often do not inform their patients that constipation can be a side effect of opioid use and do not ask patients receiving opioids about constipation symptoms [6,9,11]. In addition, patients often do not discuss the burden of OIC with their HCPs [9]. These deficits in patient–HCP communication regarding OIC may contribute to patients being dissatisfied with their OIC treatment [6]. The Opioid-Induced Constipation in Patients with Cancer Pain in Japan (OIC-J) observational study estimated the incidence of OIC as defined by the Rome IV criteria in Japanese cancer patients after the initiation of opioid therapy for cancer pain [12]. The primary results of that study demonstrated that the Rome IV criteria identified a similar proportion of patients to the Bowel Function Index (BFI; 56% vs. 59%, respectively) [12]. In addition, there was a significantly lower incidence of OIC in patients with more bowel movements (BMs; OIC incidence in patients with >7 BMs in the past week vs. 7 BMs vs. 3–5 BMs: 37% vs. 56% vs. 69%, respectively; *p* = 0.0008) [12]. Patient self-assessment data in the OIC-J study also demonstrated that Patient Assessment of Constipation Symptoms (PAC-SYM) and Patient Assessment of Constipation Quality of Life (PAC-QOL) scores of patients with OIC worsened significantly from baseline compared with patients without OIC by most diagnostic criteria used [13]. In addition, 54% of patients and ~40% of HCPs reported that OIC affected pain management; despite this, patients were generally satisfied with their OIC treatment [13]. These observations highlight a significant unmet need in the treatment of OIC. Newer agents, such as peripherally acting μ-opioid-receptor antagonists (PAMORAs), have the potential to improve patient outcomes because they provide a more targeted treatment than laxatives [14,15,16]. The PAMORA naldemedine was developed for the treatment of OIC and received approval in Japan in 2017 [17].

This post hoc analysis of the OIC-J study was conducted to further evaluate the associations between the Rome IV criteria and other measures used to assess OIC, including BFI, frequency of spontaneous bowel movements (SBMs), and physician’s diagnosis; correlation between patient demographics and OIC onset, adjusted for BM frequency prior to opioid therapy; the impact of OIC on pain treatment; and the impact of patient–HCP communication on patient satisfaction with OIC treatment.

## 2. Materials and Methods

### 2.1. Study Design and Patient Population

The study design details of the OIC-J study (UMIN000025864) have been reported previously [12]. In brief, OIC-J was a multicenter, prospective, observational cohort study involving patients aged ≥20 years with stable cancer who were initiating strong opioid therapy for cancer pain at 28 medical institutions from 5 January 2017 to 31 January 2018. Enrolled patients had an Eastern Cooperative Oncology Group performance status (ECOG PS) ≤2 and no constipation, defined as ≥3 BMs within 7 days of enrollment. Patients who had any current or a previous condition or intervention that could affect the structure or function of the gastrointestinal tract were excluded.

### 2.2. Study Endpoints and Analysis

This post hoc analysis used data collected during the OIC-J study period to evaluate the incidence of OIC based on the Rome IV diagnostic criteria [18] (primary endpoint; Supplemental Table S1). Secondary endpoints of the OIC-J study included measurements of the incidence of OIC based on BFI [19], number of SBMs per week, and physician’s diagnosis. Additionally, this analysis evaluated the impact of OIC on the effectiveness of pain treatment; the correlation between OIC-onset and patient demographics, adjusted for BM frequency prior to opioid treatment; and the correlation between patient satisfaction with OIC treatment and degree of patient–HCP communication regarding BM symptoms.

Patients recorded their bowel habits in paper diaries for 14 days following initiation of opioid analgesic medication. Items recorded were date and time of each BM, stool form according to the Bristol Stool Scale [20], presence or absence of the feeling of incomplete evacuation, degree of straining, and sensation of anorectal obstruction/blockage. Treatments for constipation were permitted during the study period.

### 2.3. Study-Specific Questionnaires

Information regarding the study-specific questionnaires has been previously described [13]. In brief, patient awareness of their OIC was assessed using the question, “After the start of opioid analgesic medication, did you think that you began having constipation because of influence of the opioid analgesic medication?” Patients, physicians, and nurses were asked to rate their satisfaction with the laxative medication administered during the study period, with available answers being “very satisfied”, “somewhat satisfied”, “minimally satisfied”, and “not at all satisfied.” The answers included an option for no laxative medication used during the study period. Patients were also asked about the degree of communication they had with their HCP regarding their BM symptoms, with available answers being “sufficiently communicated”, “essentially communicated”, “only briefly communicated”, and “not much communication”.

### 2.4. Statistical Analyses

Two populations were defined for analysis. The full analysis set (FAS) 1 population was defined as all enrolled patients, except those with ethical guideline violations, those with an observation period of <4 days, and those who did not take opioids during the observation period. The FAS 2 inpatient population was defined as all patients in the FAS 1 population with an observation period of ≥7 days who were hospital inpatients during the study observation period. For the association between the Rome IV criteria and BFI measurements in the FAS 2 population, the proportion of patients with OIC determined by the BFI (score > 28.8) among those patients with or without OIC determined by Rome IV criteria was calculated. For the association between the Rome IV criteria and SBM frequency, the proportion of patients with OIC determined by SBM (<3 per week) among those patients with or without OIC determined by Rome IV criteria was calculated. For the association between the Rome IV criteria and physician’s diagnosis, the proportion of patients with OIC determined by physician’s diagnosis among those patients with or without OIC determined by Rome IV criteria was calculated. Calculations were performed for the overall 2-week observation period. To assess the correlation between OIC onset and patient demographics in the FAS 1 population, the odds ratio (OR) and the corresponding 95% confidence interval (CI) were calculated using a logistic regression model with stool frequency at 1 week before the observation period used as a covariate. The impact of OIC on the effectiveness of pain treatment on FAS 2 inpatients was determined by estimating the proportion of patients with sustained pain control in patients with or without OIC as determined by Rome IV criteria. Sustained pain control was defined as no increase in opioid dosage and a Numerical Pain Rating Scale (NRS) score of ≤3 for 3 consecutive days. The correlation between the degree of patient satisfaction with their OIC treatment and the degree of patient–HCP communication about BM symptoms was determined using cross-tabulation. The population used for this analysis were those patients in the FAS 1 population who demonstrated OIC awareness in their answers to the questionnaire. ORs and 95% CIs were calculated using a logistic regression model. All statistical tests were performed on observed values, with a two-sided significance level of 0.05. No multiplicity tests were performed. SAS software for Windows, Version 9.4 (SAS Institute Inc., Cary, NC, USA) was used for data analysis.

## 3. Results

### 3.1. Patients

Of the 220 patients enrolled in the OIC-J study, 212 patients were included in the FAS 1 population. Of these 212 patients, 208 patients that had an observation period of ≥7 days and were hospital inpatients during the study observation period were included in the FAS 2 inpatient population. The doses and route of opioid administration were 22 ± 15 mg/day, oral (96%) and transdermal (8%) in FAS1 population and were 22 ± 15 mg/day, oral (96%) and transdermal (7%) in FAS 2 population (Supplement Table S2).

### 3.2. Association between Rome IV Criteria and the BFI

For correlations between the Rome IV criteria and BFI, 85 patients in the FAS 2 population had OIC according to the Rome IV criteria, whereas 117 patients had OIC according to BFI (Table 1). A total of 69 patients (81.2%) who were identified as OIC-positive by the Rome IV criteria were also identified as OIC-positive by the BFI (Table 1). Of the 106 patients without OIC based on Rome IV criteria, 48 patients (45.3%) were identified as having OIC by the BFI. The sensitivity and specificity of BFI for identifying OIC were 81.2% and 54.7%, respectively (Table 1).

### 3.3. Association between Rome IV Criteria and SBMs or Physician’s Diagnosis

For correlations between the Rome IV criteria and SBMs, 92 patients in the FAS 2 population had OIC according to the Rome IV criteria and 66 patients had OIC based on frequency of SBMs (Table 1). A total of 49 patients (53.3%) who were identified as OIC-positive by the Rome IV criteria were also identified as OIC-positive by SBM. The sensitivity and specificity of SBMs for identifying OIC were 53.3% and 85.3%, respectively (Table 1).

For correlations between the Rome IV criteria and physician’s diagnosis, 85 patients in the FAS 2 population were diagnosed with OIC according to the Rome IV criteria, whereas 124 patients had OIC based on physician’s diagnosis (Table 1). A total of 71 patients (83.5%) who were identified as OIC-positive by the Rome IV criteria were also identified as OIC-positive by physician’s diagnosis (Table 1). The sensitivity and specificity of a physician’s diagnosis for OIC were 83.5% and 49.5%, respectively, and were comparable to values observed with BFI (Table 1).

### 3.4. Correlation between OIC Onset and Patient Demographics

With adjustment for baseline BMs as a covariate in the FAS 1 population, there was a correlation between the onset of OIC and age <65 vs. ≥65 years (OR = 0.510, 95% CI: 0.267–0.977) and the presence or absence of comorbidities (OR = 0.443, 95% CI: 0.221–0.885; Table 2). No correlations were observed for other baseline characteristics, including sex, ECOG PS, anticancer medications, use of laxatives, and hospital admission status.

### 3.5. Sustainable Pain Control

In the FAS 2 population, the proportion of inpatients with sustainable pain control at Week 2 was 60.0% (95% CI 45.2–73.6%) in patients with OIC (*n* = 50) and 67.2% (95% CI 54.0–78.7%) in patients without OIC (*n* = 61). The time to reach sustainable pain control was similar between groups. By Day 9, more than 50% of patients had sustained pain control regardless of OIC status (Table 3).

### 3.6. Correlation between Patient Satisfaction with OIC Treatment and Degree of Patient–HCP Communications Regarding BM Symptoms

According to patient assessment, there was a significant correlation between an adequate level of patient–HCP communication and being satisfied with the OIC treatment provided (OR = 9.538, 95% CI:1.577–57.681; Table 4). A total of 62 patients were either “very satisfied” or “satisfied” with both OIC treatment and the degree of communication of BM symptoms with HCPs.

## 4. Discussion

The results of this post-hoc analysis demonstrated an association between the Rome IV criteria and BFI for identifying patients with OIC. In addition, the sensitivity and specificity of a physician’s diagnosis for OIC were comparable to that of BFI. Both age (<65 years old vs. ≥65 years old) and comorbidities (presence vs. absence) were found to correlate with OIC onset. Results also demonstrated that the presence of OIC did not have a significant impact on pain control and an adequate patient-reported level of communication with the patient’s HCP was significantly associated with treatment satisfaction.

The primary results of the OIC-J observational study demonstrated good agreement between the Rome IV criteria and BFI in estimating the proportion of patients with OIC [12], and the results of the present post-hoc analysis confirm this concordance. In the present analysis, the BFI demonstrated good sensitivity and specificity (81.2% of patients were OIC-positive by both BFI and Rome IV criteria and 54.7% of patients were OIC-negative by both the BFI and Rome IV criteria). These results are notable given that the BFI is easy to use and may, therefore, be more suitable for use in day-to-day clinical practice [19]. In addition, the BFI is expected to be used as a screening tool [19,21,22,23] rather than as a replacement of the Rome IV criteria. There are other differences between the BFI and Rome IV criteria that may impact their clinical utility. For example, a real-world study found that the Rome IV criteria may underestimate the number of patients with less severe OIC, because these patients do not demonstrate a complete array of symptoms [6]. In addition, although the Rome IV criteria assesses OIC in greater detail [18], the three-item BFI has been validated in OIC [21,22,23] and is recommended by expert consensus as a practical tool for the assessment of OIC in a diverse group of patients [24].

Additional analyses examined correlations between the Rome IV criteria and SBM frequency and physician’s diagnosis. Although measurement by SBM resulted in high specificity for identifying OIC, it demonstrated low sensitivity, with almost half (46.7%) of the patients identified as OIC-positive by Rome IV criteria not identified by evaluation of SBMs. This is not surprising given that infrequent SBMs represent a single symptom of OIC that is not always the most prevalent or bothersome to patients, and highlights a limitation of using a single parameter for identifying patients with OIC [25]. The sensitivity and specificity of a physician’s diagnosis for identifying patients with OIC, however, was comparable to that of BFI, providing some evidence that supports the use of BFI as a substitute for physician’s diagnosis in daily practice.

The association between patient characteristics and the onset of OIC varies in the literature, with some studies reporting an effect of age and sex [26] whereas others do not [27]. The results from the present analysis indicate that the presence of comorbidities confers a lower risk of OIC, which agree with the primary results of the OIC-J study [12]. In the present analysis, age ≥65 years was associated with a decreased risk of OIC compared with age <65 years, which was similar to another study that found a lower risk of severe constipation in older patients [28]. Although other studies have reported that age ≥50 years was associated with OIC [26], we did not observe an association of age with OIC when using different age ranges in the primary analysis of OIC-J [12]. Further investigation of potential relationships between baseline patient characteristics and OIC is therefore warranted.

A previous analysis found that more than 50% of patients who participated in the OIC-J study reported that OIC impacted their pain management [13], a similar result to those reported in other studies [8,10]. Investigation into sustained pain control according to presence or absence of OIC in the present analysis demonstrated that a numerically lower proportion of patients achieved sustainable pain control in patients with OIC compared with patients without OIC. This may be due, in part, to patients refusing opioids due to OIC, such that pain control was not adequately achieved.

The significant correlation between adequate patient–HCP communication and treatment satisfaction found in this study is supported by other studies reporting both patient dissatisfaction with treatment and lack of HCP communication [6], and by studies suggesting that lack of patient–HCP communication leads to suboptimal and/or difficult self-management of both pain and OIC symptoms [6,7,9,29]. In this study, a correlation was not observed between the level of patient satisfaction with OIC treatment and the level of symptom sharing between patients and HCPs, as reported by physicians and nurses. However, this result should be interpreted with caution because the frequency of some patient responses in the questionnaires was low.

The limitations of this analysis are (1) including the short observation period from opioid initiation and patient bias that may have been introduced due to the exclusion of patients with <3 BMs and patients being in generally good condition, (2) the small sample size and the study being a post hoc exploratory analysis. (3) This study used questionnaires for obtaining the self-reported data which may have introduced bias due to the accuracy. (4) This study recorded data only for the first 14 days after initiation of OIC treatment. Further study would be required to examine the long-term effects of OIC.

## 5. Conclusions

The results of this post-hoc analysis demonstrate that use of the BFI for the assessment of OIC is a valid approach in real-world settings. The presence of OIC did not have an impact on pain treatment and did not significantly impact the proportion of patients with sustained pain control, whereas age (<65 or ≥65 years) and comorbidities (presence or absence) were correlated with OIC onset. In addition, patient reporting of adequate communication with their HCP regarding bowel symptoms was significantly associated with patient satisfaction with OIC treatment, emphasizing the importance of good patient–HCP communication in the effective management of OIC. It may be beneficial for healthcare providers other than clinicians (e.g., care workers, nonmedical professionals) to be able to administer an easy-to-use BFI scale. Additional studies are warranted to verify the use of BFI as a screening tool for OIC in patients with cancer pain.

## Figures and Tables

**Table 1:** Associations Between the Rome IV Criteria and BFI, SBMs, and Physician’s Diagnosis with Respect to the Identifying Patients with OIC (FAS 2 Population).

	Onset of OIC (Rome IV), *n* (%)		
	OIC (−)	OIC (+)	Total, *n*
Onset of OIC (BFI), *n* (%)			
OIC (−)	58 (54.7)	16 (18.8)	74
OIC (+)	48 (45.3)	69 (81.2)	117
Total, *n*	106	85	191
Onset of OIC (<3 SBMs per week), *n* (%)			
OIC (−)	99 (85.3)	43 (46.7)	142
OIC (+)	17 (14.7)	49 (53.3)	66
Total, n	116	92	208
Onset of OIC (physician’s diagnosis), *n* (%)			
OIC (−)	52 (49.5)	14 (16.5)	66
OIC (+)	53 (50.5)	71 (83.5)	124
Total, *n*	105	85	190

BFI, Bowel Function Index; FAS, full analysis set; OIC, opioid-induced constipation; SBM, spontaneous bowel movement.

**Table 2:** Correlation Between OIC and Patient Demographics, Adjusted for BMs in the Past Week (FAS 1 Population).

Parameter	OR (95% CI)
Sex Male ^a^ Female	0.867 (0.473–1.590)
Age <65 years ^a^ ≥65 years	0.510 (0.267–0.977)
Admission status Inpatient ^a^ Outpatient	0.930 (0.528–1.639)
ECOG PS 0 ^a^ 1 2	0.715 (0.359–1.426) 1.012 (0.419–2.442)
Anticancer medications No ^a^ Yes	1.565 (0.887–2.760)
Rescue laxatives ≤24 h before enrollment No ^a^ Yes	2.041 (0.533–7.820)
Regular laxatives before enrollment No ^a^ Yes	0.797 (0.422–1.507)
Comorbidities No ^a^ Yes	0.443 (0.221–0.885)
Hypertension No ^a^ Yes	1.458 (0.725–2.933)
Diabetes No ^a^ Yes	0.617 (0.235–1.617)

^a^ Reference value for OR calculation. BM, bowel movement; CI, confidence interval; ECOG PS, Eastern Cooperative Oncology Group performance status; FAS, full analysis set; OIC, opioid-induced constipation; OR, odds ratio.

**Table 3:** Days to Sustained Pain Control in Patients Receiving Opioid Analgesics for Cancer Pain (FAS 2 Inpatient Population).

Time From Opioid Initiation (Days)	Patients without OIC			Patients with OIC		
	*n*	Sustained Pain Control		*n*	Sustained Pain Control	
		*n* (%)	95% CI		*n* (%)	95% CI
1	61	0	0.0–5.9	50	0	0.0–7.1
2	61	0	0.0–5.9	50	0	0.0–7.1
3	61	8 (13.1)	5.8–24.2	50	4 (8.0)	2.2–19.2
4	61	22 (36.1)	24.2–49.4	50	9 (18.0)	8.6–31.4
5	61	24 (39.3)	27.1–52.7	50	12 (24.0)	13.1–38.2
6	61	27 (44.3)	31.5–57.6	50	19 (38.0)	24.7–52.8
7	61	30 (49.2)	36.1–62.3	50	22 (44.0)	30.0–58.7
8	61	30 (49.2)	36.1–62.3	50	25 (50.0)	35.5–64.5
9	61	32 (52.5)	39.3–65.4	50	26 (52.0)	37.4–66.3
10	61	35 (57.4)	44.1–70.0	49	25 (51.0)	36.3–65.6
11	60	37 (61.7)	48.2–73.9	49	27 (55.1)	40.2–69.3
12	60	37 (61.7)	48.2–73.9	49	27 (55.1)	40.2–69.3
13	60	39 (65.0)	51.6–76.9	49	27 (55.1)	40.2–69.3
14	60	40 (66.7)	53.3–78.3	49	29 (59.2)	44.2–73.0

CI, confidence interval; FAS, full analysis set; OIC, opioid-induced constipation.

**Table 4:** Correlation Between Patient Satisfaction with OIC Treatment and Degree of Patient–HCP Communication Regarding BM Symptoms (FAS 1 Population with OIC Awareness).

		Degree of Patient Satisfaction with OIC Treatment, *n*		OR (95% CI)
		Very Satisfied/Satisfied	Not Very Satisfied/Not Satisfied	
Degree of patient–HCP communication regarding bowel movement symptoms, *n*				
Patient’s assessment (*n* = 81)	Very satisfied/satisfied	62	13	
	Not very satisfied/not satisfied	2	4	9.538 (1.577–57.681)
Physician’s assessment (*n* = 79)	Very satisfied/satisfied	62	16	
	Not very satisfied/not satisfied	0	1	NE (NE)
Nurse’s assessment (*n* = 56)	Very satisfied/satisfied	42	10	
	Not very satisfied/not satisfied	2	2	4.200 (0.526–33.543)

BM, bowel movement; CI, confidence interval; FAS, full analysis set; HCP, healthcare professional; NE, not estimable; OIC, opioid-induced constipation; OR, odds ratio.

## Data Availability

Data not available/the data that have been used are confidential.

## Supplementary Materials

The following are available online at https://www.mdpi.com/article/10.3390/jcm10184193/s1, Table S1: OIC Diagnostic Criteria Used in This Study Based on the Rome IV Diagnostic Criteria; available via link from the published article online; Table S2: Summary of opioid regimens during the study.

## References

[1] Fallon M., Giusti R., Aielli F., Hoskin P., Rolke R., Sharma M., Ripamonti C.I., ESMO Guidelines Committee (2018). Management of cancer pain in adult patients: ESMO Clinical Practice Guidelines. Ann. Oncol. Off. J. Eur. Soc. Med. Oncol..

[2] Farmer A.D., Drewes A.M., Chiarioni G., de Giorgio R., O’Brien T., Morlion B., Tack J. (2019). Pathophysiology and management of opioid-induced constipation: European expert consensus statement. United Eur. Gastroenterol. J..

[3] Rumman A., Gallinger Z.R., Liu L.W.C. (2016). Opioid induced constipation in cancer patients: Pathophysiology, diagnosis and treatment. Expert Rev. Qual. Life Cancer Care.

[4] Dorn S., Lembo A., Cremonini F. (2014). Opioid-induced bowel dysfunction: Epidemiology, pathophysiology, diagnosis, and initial therapeutic approach. Am. J. Gastroenterol. Suppl..

[5] O’Brien T., Christrup L.L., Drewes A.M., Fallon M.T., Kress H.G., McQuay H.J., Mikus G., Morlion B.J., Perez-Cajaraville J., Pogatzki-Zahn E. (2017). European Pain Federation position paper on appropriate opioid use in chronic pain management. Eur. J. Pain.

[6] Andresen V., Banerji V., Hall G., Lass A., Emmanuel A.V. (2018). The patient burden of opioid-induced constipation: New insights from a large, multinational survey in five European countries. United Eur. Gastroenterol. J..

[7] Epstein R.S., Teagarden J.R., Cimen A., Sostek M., Salimi T. (2017). When people with opioid-induced constipation speak: A patient survey. Adv. Ther..

[8] Gupta A., Coyne K.S., Datto C., Venuti C. (2018). The burden of opioid-induced constipation in younger patients with chronic noncancer pain. Pain Med..

[9] LoCasale R.J., Datto C., Wilson H., Yeomans K., Coyne K.S. (2016). The burden of opioid-induced constipation: Discordance between patient and health care provider reports. J. Manag. Care Spec. Pharm..

[10] Coyne K.S., Sexton C., LoCasale R.J., King F.R., Margolis M.K., Ahmedzai S.H. (2016). Opioid-induced constipation among a convenience sample of patients with cancer pain. Front. Oncol..

[11] Vallerand A.H., Hendry S., Baldys E., Hu Y., Datto C. (2019). Analysis of patient-provider interactions regarding the burden and treatment of opioid-induced constipation in adults with chronic noncancer pain. Pain Med..

[12] Tokoro A., Imai H., Fumita S., Harada T., Noriyuki T., Gamoh M., Akashi Y., Sato H., Kizawa Y. (2019). Incidence of opioid-induced constipation in Japanese patients with cancer pain: A prospective observational cohort study. Cancer Med..

[13] Fumita S., Imai H., Harada T., Noriyuki T., Gamoh M., Akashi Y., Sato H., Kizawa Y., Tokoro A. (2020). Patients’ self-assessment of the symptoms and impact of opioid-induced constipation: Results from a prospective observational cohort study of Japanese patients with cancer. J. Pain Symptom Manag..

[14] Brenner D.M., Chey W.D. (2014). An evidence-based review of novel and emerging therapies for constipation in patients taking opioid analgesics. Am. J. Gastroenterol. Suppl..

[15] Pergolizzi J.V., Raffa R.B., Pappagallo M., Fleischer C., Pergolizzi J., Zampogna G., Duval E., Hishmeh J., LeQuang J.A., Taylor R. (2017). Peripherally acting μ-opioid receptor antagonists as treatment options for constipation in noncancer pain patients on chronic opioid therapy. Patient Prefer. Adherence.

[16] Lemaire A., Pointreau Y., Narciso B., Piloquet F., Braniste V., Sabaté J. (2021). Effectiveness of naloxegol in patients with cancer pain suffering from opioid-induced constipation. Support Care Cancer.

[17] Pharmaceuticals and Medical Devices Agency Report on the Deliberation Results: Symproic Tablets 0.2 mg. https://www.pmda.go.jp/files/000222647.pdf.

[18] Lacy B.E., Mearin F., Chang L., Chey W.D., Lembo A.J., Simren M., Spiller R. (2016). Bowel disorders. Gastroenterology.

[19] Ueberall M.A., Müller-Lissner S., Buschmann-Kramm C., Bosse B. (2011). The Bowel Function Index for evaluating constipation in pain patients: Definition of a reference range for a non-constipated population of pain patients. J. Int. Med. Res..

[20] Lewis S.J., Heaton K.W. (1997). Stool form scale as a useful guide to intestinal transit time. Scand. J. Gastroenterol..

[21] Abramowitz L., Béziaud N., Caussé C., Chuberre B., Allaert F.A., Perrot S. (2013). Further validation of the psychometric properties of the Bowel Function Index for evaluating opioid-induced constipation (OIC). J. Med. Econ..

[22] Rentz A.M., Yu R., Muller-Lissner S., Leyendecker P. (2009). Validation of the Bowel Function Index to detect clinically meaningful changes in opioid-induced constipation. J. Med. Econ..

[23] Rentz A.M., van Hanswijck de Jonge P., Leyendecker P., Hopp M. (2011). Observational, nonintervention, multicenter study for validation of the Bowel Function Index for constipation in European countries. Curr. Med. Res. Opin..

[24] Argoff C.E., Brennan M.J., Camilleri M., Davies A., Fudin J., Galluzzi K.E., Gudin J., Lembo A., Stanos S.P., Webster L.R. (2015). Consensus recommendations on initiating prescription therapies for opioid-induced constipation. Pain Med..

[25] Poulsen J.L., Brock C., Olesen A.E., Nilsson M., Drewes A.M. (2015). Evolving paradigms in the treatment of opioid-induced bowel dysfunction. Ther. Adv. Gastroenterol..

[26] Ducrotté P., Milce J., Soufflet C., Fabry C. (2017). Prevalence and clinical features of opioid-induced constipation in the general population: A French study of 15,000 individuals. United Eur. Gastroenterol. J..

[27] Clark K., Lam L.T., Talley N.J., Phillips J.L., Currow D.C. (2017). Identifying factors that predict worse constipation symptoms in palliative care patients: A secondary analysis. J. Palliat Med..

[28] Corli O., Roberto A., Bennett M.I., Galli F., Corsi N., Rulli E., Antonione R. (2018). Nonresponsiveness and susceptibility of opioid side effects related to cancer patients’ clinical characteristics: A post-hoc analysis. Pain Pract..

[29] Whitman C.B., Reid M.W., Arnold C., Patel H., Ursos L., Sa’adon R., Pourmorady J., Spiegel B.M. (2015). Balancing opioid-induced gastrointestinal side effects with pain management: Insights from the online community. J. Opioid Manag..

