# Supplementary material for: Opioid-Induced Constipation in Patients with Cancer Pain in Japan (OIC-J Study): A Post Hoc Analysis
Source: J Clin Med. 2021 Sep 16;10(18):4193. doi: 10.3390/jcm10184193 (PMC8472365; doi:10.3390/jcm10184193)
Supplement: Supplementary file 1 [file jcm-10-04193-s001.zip › jcm-1319348-supplementary.pdf]

Supplementary Table S1. OIC Diagnostic Criteria Used in This Study Based on the Rome IV Diagnostic Criteria; available via link from the published article online.

	A condition is assessed as OIC when 2 or more of the following are met:
1	Straining during more than 25% of defecations
2	Lumpy or hard stools (Bristol Stool Form scale 1–2) more than 25% of defecations
3	Sensation of incomplete evacuation more than 25% of defecations
4	Sensation of anorectal obstruction/blockage more than 25% of defecations
5	Fewer than 3 spontaneous bowel movements per week

OIC, opioid-induced constipation.

Information from Lacy et al. *Gastroenterology*. 2016;150:1393-1407.

Supplementary Table S2. Summary of opioid regimens during the study.

**Parameter**	**FAS 1 (n = 212)**	**FAS 2 (n = 208)**
Prescribed opioids , n (%)		
Regular use	193 (91)	189 (91)
Oxycodone	150 (71)	148 (71)
Morphine	16 (8)	14 (7)
Fentanyl	15 (7)	14 (7)
Tapentadol	10 (5)	10 (5)
Hydromorphone	13 (6)	13 (6)
Rescue use	152 (72)	148 (71)
Oxycodone	118 (56)	116 (56)
Morphine	24 (11)	22 (11)
Fentanyl	2 (1)	2 (1)
Hydromorphone	9 (4)	9 (4)
Mean doses[1)], mg/day (SD)		
Overall	22 (15)	22 (15)
Regular use	19 (12)	20 (12)
Rescue use	6 (6)	6 (6)
Route, n (%)		

Oral	203 (96)	199 (96)
Injection	17 (8)	15 (7)
Transdermal	16 (8)	15 (7)
Others	3 (1)	3 (1)

Daily morphine equivalent dose per patient, FAS: full analysis set, SD: standard deviation
